# Degradation of mitochondrial alternative oxidase in the appendices of *Arum maculatum*

**DOI:** 10.1042/BCJ20200515

**Published:** 2020-09-17

**Authors:** Kikukatsu Ito, Takafumi Ogata, Takanari Seito, Yui Umekawa, Yusuke Kakizaki, Hiroshi Osada, Anthony L. Moore

**Affiliations:** 1 Faculty of Agriculture, Iwate University, 3-18-8 Ueda, Morioka, Iwate 020-8550, Japan; 2 Agri-Innovation Center, Iwate University, 3-18-8 Ueda, Morioka, Iwate 020-8550, Japan; 3 United Graduate School of Agricultural Science, Iwate University, 3-18-8 Ueda, Morioka, Iwate 020-8550, Japan; 4 Department of Electrical and Electronic Engineering, Faculty of Science and Engineering, Iwate University, 4-3-5 Ueda, Morioka, Iwate 020-8551, Japan; 5 Department of Biochemistry and Biomedicine, School of Life Sciences, University of Sussex, Brighton BN1 9QG, U.K.

**Keywords:** alternative oxidase, mitochondria, temperature, thermogenic plants

## Abstract

Cyanide-resistant alternative oxidase (AOX) is a nuclear-encoded quinol oxidase located in the inner mitochondrial membrane. Although the quality control of AOX proteins is expected to have a role in elevated respiration in mitochondria, it remains unclear whether thermogenic plants possess molecular mechanisms for the mitochondrial degradation of AOX. To better understand the mechanism of AOX turnover in mitochondria, we performed a series of *in organello* AOX degradation assays using mitochondria from various stages of the appendices of *Arum maculatum*. Our analyses clearly indicated that AOX proteins at certain stages in the appendices are degraded at 30°C, which is close to the maximum appendix temperature observed during thermogenesis. Interestingly, such temperature-dependent protease activities were specifically inhibited by E-64, a cysteine protease inhibitor. Moreover, purification and subsequent nano LC–MS/MS analyses of E-64-sensitive and DCG-04-labeled active mitochondrial protease revealed an ∼30 kDa protein with an identical partial peptide sequence to the cysteine protease 1-like protein from *Phoenix dactylifera*. Our data collectively suggest that AOX is a potential target for temperature-dependent E-64-sensitive cysteine protease in the appendices of *A. maculatum*. A possible retrograde signalling cascade mediated by specific degradation of AOX proteins and its physiological significance are discussed.

## Introduction

Thermogenesis in reproductive organs occurs in various seed plants including *Araceae, Aristolochiaceae, Annonaceae, Cycadaceae, Cyclanthaceae, Magnoliaceae, Nelumbonaceae* and *Nymphaeaceae* [1]. Since the seminal report in 1955 [2] that mitochondria in the spadix of *Arum maculatum*, a thermogenic plant in *Araceae*, exhibit cyanide-insensitive respiration, significant progress has been made in establishing the structure-function relationships of cyanide-insensitive alternative oxidase (AOX). Currently thermogenesis in plants is attributed partially to the activity of AOX in the mitochondria [3–9]. AOX is part of an alternative respiratory pathway specific to plants as well as certain fungi and protists, which acts as an electron acceptor from ubiquinol, thereby bypassing complex III [9,10]. AOX reduces oxygen to water without the establishment of a proton gradient, thereby allowing a dramatic drop in free energy between ubiquinol and oxygen which is dissipated as metabolic heat [5,9,11,12]. Respiratory analysis with mitochondria purified from thermogenic tissues has revealed a large cyanide-insensitive respiration capacity in *A. maculatum* [13–15], *Symplocarpus renifolius* [16], *Nelumbo nucifera* [17], *Dracunculus vulgaris* [18] and *Cycas revoluta* [19].

AOX is an interfacial membrane protein oriented towards the matrix side of the inner mitochondrial membrane [12,20], and crystallization of the trypanosomal AOX reveals that it is a homodimer, with each monomer comprising four short and six long α-helices arranged in an antiparallel fashion, four of which create a four-helix bundle that acts as a scaffold to bind the two iron atoms [21]. The AOX dimer is present either as a non-covalently-linked active form or a covalently-linked inactive form, with a regulatory disulfide bond between the two monomers via a conserved cysteine residue (Cys I) located towards the N-terminus of the AOX protein [22]. Moreover, once in reduced form, AOX proteins that harbour the ENV motif are activated by pyruvate [16], whereas AOX proteins with a QNT or QDC motif are pyruvate-insensitive [7,15]. In some plants, the Cys I residue of the AOX isoforms is replaced by serine, and this change results in the activation of AOX activity by succinate rather than pyruvate [17,23].

The level of AOX expression varies among non-thermogenic plant species. In potato tuber mitochondria, for example, the AOX protein level is much lower than that found in thermogenic plants. In general, AOX is considered to be expressed when the main respiratory chain is inhibited or the plant is exposed to extreme environmental stresses such as drought, excessive salinity or low temperatures [24]. Moreover, the AOX-mediated respiration pathway provides a mechanism for oxidative metabolism in the absence of ATP synthesis. Mitochondria are a source of ROS production because an electron leakage from respiratory chain components, in particular from complexes I and III, leads to the production of superoxide (O_2_^−^) from oxygen [25,26]. AOX acts to prevent the over-reduction of electron transfer chain components that leads to such ROS production [27,28]. Accordingly, abiotic and biotic conditions associated with oxidative stress could induce *AOX* gene expression, thereby maintaining cell homeostasis [24,29].

AOX has also been shown to be regulated in a retrograde manner [30–37]. For instance, the transcription factor ABI4 acts as a repressor of AOX1a, which is depressed under rotenone treatment [38,39]. Although ABI4 represents a downstream component of a mitochondrial retrograde signalling pathway, the actual sensors in the organelle and the second messengers of the signalling cascade are still not well known [40,41].

Thus, although there is a wealth of information on *AOX* gene expression under various environmental conditions and in various signalling pathways, little is known regarding the quality control of the AOX protein, such as its protein degradation in mitochondria. For instance, LC–MS/MS analyses using progressive ^15^N labelling of *Arabidopsis* cells have shown that different mitochondrial proteins turnover at widely divergent rates, with a >50-fold variation in protein degradation [42], although the AOX protein appears to have been undetectable in these analyses. The heat-shock chaperone type protein, grpE, showed the shortest half-life (31 h) whereas components of mitochondrial energy biogenesis, including cytochrome *c* oxidase 15 and NAD (P) H dehydrogenase B2, showed no appreciable turnover during 7 days of measurements.

Thermogenesis in the appendix of *A. maculatum* is developmentally regulated [43], and there are at least seven types of *AOX* transcripts (*AmAOX1a*–*AmAOX1g*) in the appendix [15]. The expression of AmAOX1e, which harbours the QNT type of pyruvate-insensitive isoform, dominates in thermogenic appendices of *A. maculatum* [15]. Because the expression of *AmAOX1e* gene transcripts is highly specific to thermogenic tissues in *A. maculatum*[15], we hypothesized that this thermogenic plant would be an ideal organism to clarify the relationship between *AOX* gene expression (i.e. levels of its transcripts and proteins) and the quality control of AOX proteins in the context of developmentally regulated thermogenesis.

To investigate whether co-ordinated *AOX* gene expression in thermogenic appendices is associated with specific turnover of the AOX protein in the mitochondria, we employed a series of *in organello* protein degradation assays using mitochondria purified from the appendices of *A. maculatum*.

## Materials and methods

### Plant materials

The appendices of *A. maculatum* plants at β-, δ-, and γ/δ periphery stages were collected on the campus of the University of Sussex, U.K..

### Thermal imaging

Thermal images were captured with an infrared colour LSD camera (TVS-600; Nippon Avionics, Tokyo, Japan) and images were analyzed for temperature determination as previously described [15,44].

### qRT-PCR and cycleave PCR analyses

Real-time qRT-PCR was performed with a Dice Thermal Cycler (TP800; Takara Bio, Otsu, Japan) as described previously [15]. Briefly, total RNA samples were isolated using the FastPure RNA kit (Takara Bio). cDNAs were then generated using a ReverTra Ace qPCR RT Kit (Toyobo, Osaka, Japan), and qRT-PCR was performed using a SYBR Green Real-time PCR Master Mix-Plus- (Toyobo). The primers used to detect the full complement of transcripts for *AmAOX1a-1g* were AmAOXallFW (5′-ggaggccatccactcctaca-3′) and AmAOXallRV (5′-acgacgtcgcgcagggtg-3′), and amplification conditions were as follows: an initial denaturing step at 95°C for 30 s; 45 amplification cycles of 95°C for 15 s, 61°C for 15 s and 72°C for 20 s; and a dissociation curve analysis at 95°C for 15 s, 60°C for 30 s and 95°C for 15 s. Cycleave PCR was performed to quantify the transcripts of ENV-, QNT- or QDT-type *AOX* genes as described in our previous study [15]. The primer sets used for *VDAC* and *ubiquitin* were as follows: AmVDAC_FW (5′-caagttcgacaccctaacaac-3′) and AmVDAC_RV (5′-agaagctgtgcgtcaactc-3′) for *VDAC*; and AmUB_FW (5′-caggtggaatcctctgacacg-3′) and AmUB_RV (5′-cgtcctccagctgcttcc-3′) for *ubiquitin*. The same amplification protocol was used for *VDAC* and ubiquitin as follows: an initial denaturing step at 95°C for 30 s; 45 amplification cycles of 95°C for 15 s, 60°C for 15 s and 72°C for 20 s; and a dissociation curve analysis at 95°C for 15 s, 60°C for 30 s and 95°C for 15 s.

### Preparation of mitochondria, respiration analysis and determination of protein concentration

Mitochondria were purified from the thermogenic appendices of *A. maculatum* as described previously [15,45]. Isolated mitochondria were stored at −30°C overnight and then at −80°C. For DCG-04 activity labelling, the stored mitochondria were treated by repeated freeze-thawing cycles and suspended in a buffer containing 2 mM TES-KOH (pH 7.5), 5 mM EDTA, 1 mM AEBSF, and 1 mM pepstatin. After centrifugation at 20 000 ***g*** at 4°C for 15 min, the supernatants were recovered (termed SUP), and precipitants (termed PPT) were resuspended in a buffer containing 0.4 M mannitol, and 10 mM MOPS-KOH (pH 7.2). Respiratory analysis was performed as described previously [15]. Protein concentrations of the isolated mitochondria were determined using the previously described BCA method [15,45].

### *In organello* protein degradation assay

Mitochondria purified from *A. maculatum* were incubated in a buffer containing 200 mM Tris–HCl, pH 7.5, 10 mM MgCl_2_, 10 mM CaCl_2_, 50 mM ATP and 0.1% Triton X-100 [46] at either 15°C or 30°C for the indicated time periods. Protease inhibitors were then added to the incubation medium at the following final concentrations: EDTA (5 mM), AEBSF (1 mM), pepstatin (1 µM) and E-64 (28 µM).

### Western blotting and immunological detection

Mitochondrial proteins were separated on SDS–PAGE gels and transferred to polyvinylidene difluoride (PVDF) membranes as described previously [15,45]. Membranes were then treated with primary antibodies against AOX (Agrisera, Vännäs, Sweden), Hsp60 or VDAC (a gift from Dr. Thomas Elthon, University of Nebraska, Lincoln, U.S.A.). Densitometric analysis of immunologically detected AmAOX proteins was carried out within the quantitative dynamic range of a CS Analyzer 3.0 (ATTO, Tokyo, Japan).

### Detection of carbonylated proteins in the mitochondria

Carbonylated proteins were detected using Oxidized Protein Western Blot Kit (MitoSciences, OR, U.S.A.) in accordance with the manufacturer's instructions. Densitometric analysis of the signals was carried out using the ImageJ Software (version 1.51).

### Activity labelling with the biotinylated probe DCG-04

Activity profiling of cysteine proteases was performed with DCG-04, a biotinylated analogue of the irreversible cysteine protease inhibitor E-64 [47–49]. Briefly, mitochondrial samples were incubated in a buffer containing 5 µM DCG-04, 50 mM Na-acetate, 1 mM cysteine, 0.1% Triton X-100 at 4°C for 2 h. Proteins were separated on SDS–PAGE gels, transferred to PVDF membranes, and detected with streptavidin-horseradish peroxidase (Thermo, MA, U.S.A.).

### Affinity purification of DCG-04-reactive proteases

To purify the DCG-04-reactive proteases from mitochondria prepared from non-thermogenic appendices, 20 µl of magnetic streptavidin beads (Promega, WI, U.S.A.) was added to each reaction mixture and incubated for 16 h at 4°C. The magnetic beads were then washed with 300 µl of TBS buffer using a magnetic stand (Promega) to remove non-specifically bound proteins. Biotin-labelled proteins were recovered by boiling the magnetic beads in Laemmli SDS loading buffer and subjected to SDS–PAGE.

### Mass spectrometry

Nano LC–MS/MS analysis was performed using a LTQ orbitrap XL system (Thermo Scientific, MA, U.S.A.) as described previously [15,45]. The MASCOT Server 2.3.02 (Matrix Science, Tokyo, Japan) software was used for Mascot database searching.

### Computer simulation

Modelling and computer simulation for AOX turnover was carried out using Excel spreadsheet program (Microsoft, WA, U.S.A.).

### Statistical analysis

Results were presented as mean values ± SD. Differences between the means were tested by one-way factorial ANOVA and a Tukey's honest significant difference post hoc test using SPSS software (IBM, NY, U.S.A.). Significant differences between means were indicated by a *P* value <0.05.

## Results

### Thermogenesis of *A. maculatum* and thermogenic appendix-specific expression of *AmAOX* transcripts

**Figure 1.**
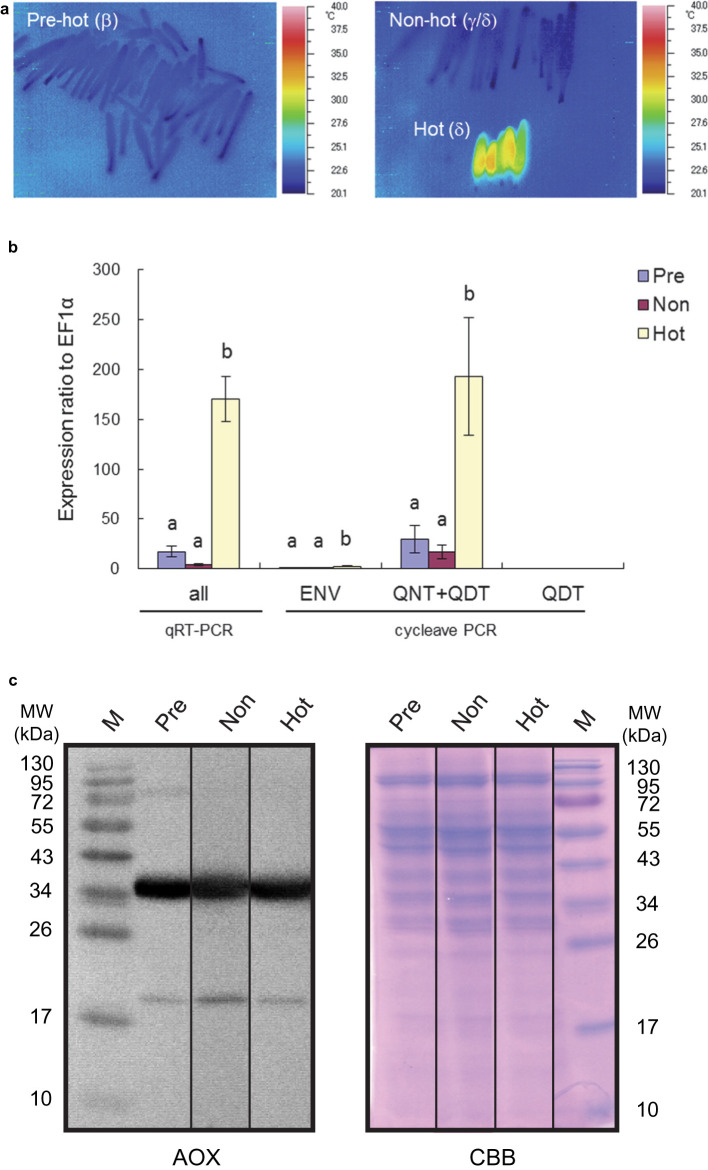
Expression of *AmAOX* transcripts and proteins in the appendices of *A. maculatum*. (**a**) Infrared thermal images of the pre-hot (β-stage), non-hot (γ/δ-stage) and hot (δ-stage) appendices used in this study. (**b**) *AmAOX* transcript levels in pre-hot, non-hot and hot stages of *A. maculatum*. These levels are shown as an expression ratio relative to *EF1a*. Expression levels analyzed by qRT-PCR and cycleave PCR are shown at the bottom of the figure. The total *AmAOX* expression level was determined using common primers that amplify all types of transcripts (*AmAOX1a*–*AmAOX1g*; denoted by ‘all’). Transcripts for QNT- and QDT-type AmAOXs were coamplified in the cycleave PCR (QNT + QDT). Values are the means ± SD (*n* = 3). Different alphabetical letters in the graph indicate significantly different values (*P *< 0.05). (**c**) Expression of AmAOX proteins in the mitochondria from various thermogenic stage appendices. Mitochondrial proteins were separated by SDS–PAGE, visualised with the Coomassie Brilliant Blue (CBB) staining (right), transferred to a polyvinylidene difluoride membrane and immunoblotted with antibodies against AOX (left). Molecular mass standards are indicated.

We have previously reported that the appendices of *A. maculatum* show intensive thermogenesis at the δ stage during the development of inflorescence, and that the maximum temperature reached ∼30°C [15]. To evaluate the expression levels of the *AmAOXs* in the appendices of this plant in our current study, the apparent δ stages of the thermogenic appendices with open but not wilted spathes were collected in the field together with the β stages of pre-thermogenic appendices with tightly closed spathes. All sampled appendices were analyzed in the laboratory with an infrared thermal camera to evaluate the level of thermogenesis (Figure 1a). No thermogenic activity was found in the appendices at the β stage, and these samples were accordingly designated ‘pre-hot’. Our thermal imaging analyses clearly showed that some appendices collected at the δ stage of inflorescence were indeed thermogenic, which were termed ‘hot’; whereas the others were not thermogenic and were denoted ‘non-hot’ (Figure 1a). Some appendices were found to be at the γ/δ periphery stage of thermogenesis, although we attempted to collect only thermogenic δ stage appendices in the field. However, because these non-hot samples acted as an experimental control by representing the physiological status at the boundary stages of thermogenesis, we decided to use them for subsequent comparative analyses of *AmAOX* gene transcript levels, and an *in organello* degradation assay of AmAOX proteins, alongside the pre-hot and hot appendices.

The *AmAOX* transcript levels were evaluated by real-time PCR using two different methods: conventional quantitative PCR (qRT-PCR), which amplified all *AmAOX* transcripts and cycleave PCR, which specifically amplified the ENV, QNT + QDT and QDT types of *AmAOX* transcripts. *AmAOX* transcripts showed significantly higher expression levels in hot appendices by both qRT-PCR and cycleave PCR (Figure 1b). Our qRT-PCR analysis revealed that *AmAOX* transcripts were ∼7–8-times more abundant in hot appendices than in pre-hot samples. The expression of transcripts encoding the QNT type of AmAOX (AmAOX1e) was significantly higher in hot appendices, supporting the notion raised in our previous report that AmAOX1e is the major AOX molecule expressed in thermogenic appendices of *A. maculatum* [15]. As the temperature rises to ∼30°C in hot appendices, it could be speculated that this is a trigger for the induction of general gene transcription, including *AmAOX1e*, in the appendices. To determine whether gene transcripts other than *AmAOX1e* are induced in the thermogenic appendices of *A. maculatum*, expression analysis for voltage-dependent anion channel (VDAC) and ubiquitin genes was conducted using the same RNA samples from pre-hot, non-hot and hot appendices that were used in the *AmAOX1e* expression analysis. VDAC has a role in regulating metabolic and energetic flux across the outer mitochondrial membrane and its gene expression is reflective of mitochondrial metabolism [50]. Ubiquitin is frequently used as a housekeeping gene in expression analysis [51]. The *VDAC* transcript levels between pre-hot and hot appendices were almost the same, whereas those in non-thermogenic appendices were significantly lower than in the other samples (Supplementary Figure S1). Considering that *VDAC* expression represents mitochondrial metabolism in the plant cell, these results suggest that there were some post-thermogenic appendices in our samples that had been collected in the field as non-hot appendices. The *ubiquitin* transcript levels were found to be consistent across pre-hot, non-hot and hot appendices (Supplementary Figure S1). Our gene expression analysis of various stage appendices of *A. maculatum* thus suggested that an increased temperature produced by thermogenesis has little effect on the accumulation of mRNAs for *VDAC* and *ubiquitin*, but that there is a thermogenic stage-specific mechanism that strongly induces the gene transcription of *AmAOX1e*.

### Expression and temperature-dependent degradation of AmAOX proteins in mitochondria

We surmised that if there was a correlation between the *AmAOX* transcript and protein levels in the appendices of *A. maculatum*, a higher accumulation of the AmAOX protein in hot appendices would be found because *AmAOX1e* transcripts are abundantly expressed under these conditions. However, by non-reducing SDS–PAGE and subsequent western analyses of mitochondria from pre-hot, non-hot and hot appendices, we observed that AmAOX proteins are present as reduced non-covalently associated dimers at similar expression levels in the mitochondria in all cases (Figure 1c). Moreover, the major proteins stained by CBB were similar regardless of the developmental stage (Figure 1c).

### Temperature-dependent degradation of AmAOX proteins in the mitochondria

**Figure 2.**
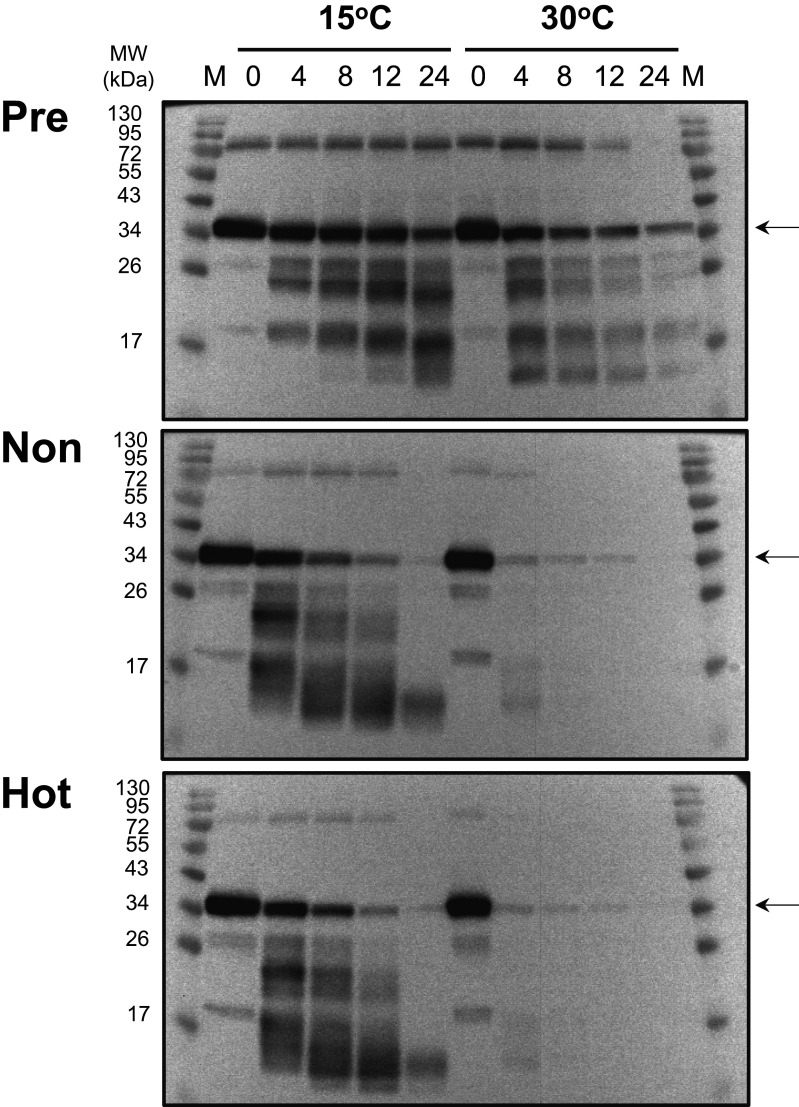
Degradation of AmAOX proteins in mitochondria purified from appendices at various developmental stages in *A. maculatum*. Mitochondrial proteins were incubated at either 15°C or 30°C in a buffer containing 200 mM Tris–HCl, pH 7.5, 10 mM MgCl_2_, 10 mM CaCl_2_, 50 mM ATP and 0.1% Triton-X100 for the indicated time periods (0, 4, 8, 12 and 24 h). Samples were separated by SDS–PAGE, transferred to a polyvinylidene difluoride membrane and incubated with antibodies to AOX. AmAOX positions are indicated by arrows. Molecular mass standards are also indicated.

To determine whether AmAOX proteins are degraded by an increased temperature, an *in organello* degradation assay with purified mitochondria was performed. Mitochondria prepared from pre-hot, non-hot and hot appendices, as shown in Figure 1, were treated at either 15°C or 30°C, followed by western analysis for AmAOX proteins (Figure 2). In the mitochondria from the pre-hot appendices, AmAOX proteins were slightly degraded at 15°C but much more quickly at 30°C. The degradation profiles of AmAOX proteins in the mitochondria from non-hot and hot appendices appeared to be essentially similar and more rapidly degraded than those from pre-hot appendices. In the mitochondria from the non-hot and hot appendices, AmAOX proteins were more rapidly degraded at 30°C than at 15°C. These results suggest that AmAOX proteins are substrates for mitochondrial protease(s) that show a higher degradation activity at 30°C.

### Carbonyl oxidation of mitochondrial proteins and respiration activities

Mitochondria are the main site of ROS production and the oxidation of mitochondrial proteins leads to a conformational change and/or modifications [52], which cause recognition and degradation by protease [53]. The best characterized irreversible protein oxidation reaction in mitochondria is carbonylation, which is regarded as an indicator of oxidative damage to the cell [53]. Accordingly, we first investigated the carbonylation of mitochondrial proteins in pre-hot, non-hot and hot appendices (Supplementary Figure S2). Our results clearly indicated that carbonylated proteins are present in mitochondria but that the patterns were comparable between non-hot and hot appendices. These results suggested that ROS produced during thermogenesis in appendices are scavenged by anti-oxidative defence mechanisms in mitochondria, although our DNP-based analysis could not determine the molecule-specific oxidation status of the mitochondrial proteins. We thus examined the oxidation of tryptophan residues, an irreversible chemical reaction [54], in the AmAOX proteins in pre-hot, non-hot and hot appendices. By nano-LC–MS/MS analysis however, we detected no N-formylkynurenine or kynurenine oxidized residues on these proteins. In general, it is thought that oxidized proteins lose functionality because of conformational changes due to the oxidization of amino acid residues [55]. Because no tryptophan residues were found to have been oxidized in AmAOX proteins and carbonylated proteins were almost equally distributed in the mitochondria from non-hot and hot appendices, we hypothesized that the mitochondrial respiration profiles, including the AOX-mediated respiration pathway, would be similar among our analyzed mitochondria.

To test this idea, we next conducted respiratory assays using NADH as the mitochondrial substrate (Supplementary Figure S3). The respiration profiles of each mitochondrial preparation were essentially equivalent irrespective of their thermogenic status. Our data also indicated that pyruvate stimulates AOX-mediated respiration in pre-hot mitochondria, whereas no activation was evident in mitochondria from the non-hot and hot appendices. These results confirmed those of our previous study [15], in which we found that the QDT type of pyruvate-insensitive AOX (AmAOX1e) is principally expressed in mitochondria from non-hot and hot appendices, whereas the mitochondria from pre-hot appendices express the ENV type of pyruvate-sensitive AOXs (AmAOX1a-d and f).

### Characterization of protease(s) that degrade mitochondrial AmAOX proteins in mitochondria

**Figure 3.**
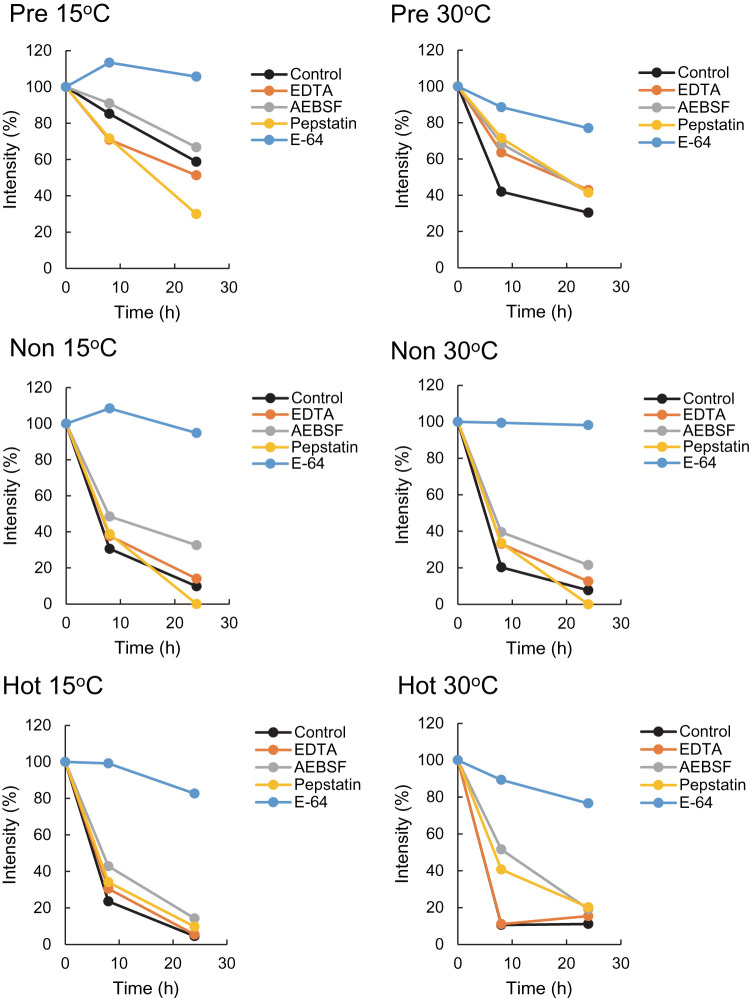
Effects of proteinase inhibitors on the degradation of AmAOX proteins in mitochondria purified from appendices at various developmental stages in *A. maculatum*. AOX protein signals shown Supplementary Figure S4 are expressed as a relative intensity following densitometric analysis as described in the Materials and methods.

To next characterize the protease(s) that degrade AmAOX proteins, we tested four types of protease inhibitor via an *in organello* protease assay: EDTA for metalloproteases, AEBSF for serine proteases, pepstatin for aspartic proteases and E-64 for cysteine proteases. To assess the specificity of the protease(s) that degrade AmAOX proteins, antibodies to Hsp60 and VDAC were also used in the western analysis (Supplementary Figure S4). We found that the addition of E-64 had apparent inhibitory effects on the degradation of AmAOX proteins in the mitochondria from pre-hot, non-hot and hot appendices at either 15°C or 30°C (Figure 3 and Supplementary Figure S4). EDTA and pepstatin produced little effect although AEBSF had a weak inhibitory effect on the degradation of AmAOX proteins. In contrast, VDAC, which localizes at the outer membrane of the mitochondria, was not degraded under our experimental conditions (Supplementary Figure S4). This suggests that E-64-sensitive protease(s) that are capable of the degradation of AmAOX proteins reside in the matrix of the mitochondria. Under these same experimental conditions, Hsp60 was not degraded in the same manner as the AmAOX proteins, although a weak degradation may have occurred over a prolonged incubation (Supplementary Figure S4).

E-64 is a mechanism-based inhibitor that specifically and irreversibly reacts with papain-like cysteine proteases [56]. Thus, our present results clearly suggest that cysteine protease(s) function in the mitochondria from *A. maculatum* and that AmAOX proteins are their natural target substrates for degradation in a temperature-dependent manner. Moreover, the E-64-sensitive degradation of AmAOX proteins proceeded faster in the mitochondria from non-hot and hot appendices at either 15°C or 30°C (Figure 2 and Supplementary Figure S4). These results further indicated that the expression of E-64-sensitive cysteine protease(s) is under the control of inflorescence development in *A. maculatum*.

### Identification of an E-64-sensitive and DCG-04-reactive cysteine protease

**Figure 4.**
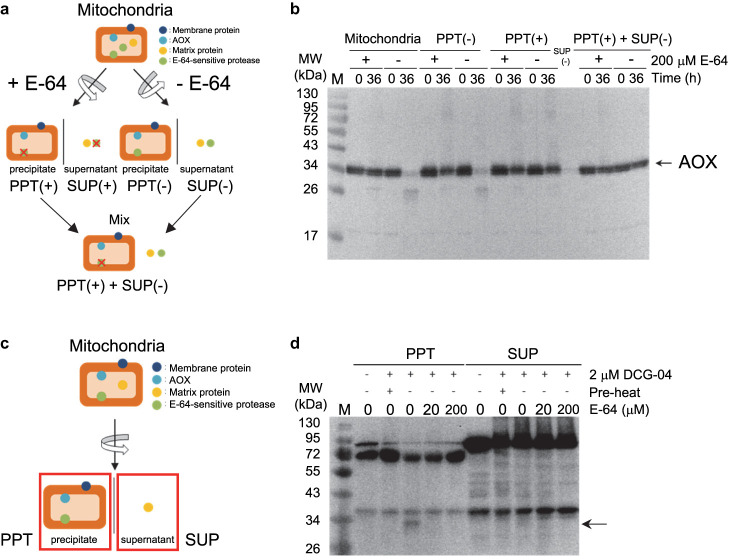
Targeting of E-64-sensitive and DCG-04-reactive proteases in mitochondria from *A. maculatum*. (**a**,**c**) Schematic representation of the experimental steps used for the detection of the AmAOX proteolysis. (**b**) E-64-sensitive protease activities. Mitochondria were incubated with or without E-64 (200 µM) at 30°C in a medium containing 200 mM Tris–HCl, pH 7.5, 10 mM MgCl_2_, 10 mM CaCl_2_, 50 mM ATP and 0.1% Triton-X100 for the indicated time periods (0 and 36 h). Samples were separated by SDS–PAGE, transferred to a polyvinylidene difluoride membrane, and incubated with antibodies to AOX. Molecular mass standards are indicated. (**d**) Detection of DCG-04 labelled active protease in the membrane fraction of the mitochondria. Mitochondria were incubated with or without DCG-04 (2 µM) for 2 h at 4°C. A pre-heated sample (100°C, 5 min) was also labelled with DCG-04 (pre-heat). Samples were separated by SDS–PAGE, transferred to a polyvinylidene difluoride membrane, and detected with streptavidin-horseradish peroxidase. The arrow on the right indicates the position of a cysteine protease specifically labelled with DCG-04. Molecular mass standards are indicated.

To examine the E-64-sensitive protease activities that degrade AOX proteins in our current experimental system, purified mitochondria were pre-treated in the presence or absence of E-64 and separated into crude membrane (PPT) and matrix (SUP) fractions (Figure 4a). Purified mitochondria were confirmed to actively degrade AOX proteins in the absence of E-64 (Figure 4b). Crude membrane fractions with no pre-treatment of E-64 (PPT (−)) also showed a similar protease activity against AOX proteins (Figure 4b), whereas this was inhibited by pre-treatment with E-64 (PPT (+)) (Figure 4b). There were no detectable AOX proteins in the matrix fraction (SUP (−)). To examine if any AOX-degrading protease activities remain in the matrix fraction, a mitochondrial membrane fraction treated with E-64 (PPT (+)) was mixed with the matrix fraction (SUP (−)) and incubated either in the presence or absence of E-64 (Figure 4b). We observed no degradation of AOX proteins in this mixture, which suggested that the E-64-sensitive protease activities localize in the mitochondrial membrane fraction.

We further characterized the E-64-sensitive protease(s) via activity profiling with the E-64 analogue DCG-04 containing a biotin tag [47]. Repeatedly freeze-thawed mitochondria were separated into crude membrane (PPT) and matrix (SUP) fractions in the absence of E-64 (Figure 4c). Profiles of DCG-04-labelled cysteine proteins were found to differ between the crude mitochondrial membrane and matrix fractions (Figure 4d), which suggested a successful separation of crude mitochondrial membrane and matrix fractions. To determine the specificity of DCG-labelled proteins, E-64 was used as a competitor. We detected a band at ∼30 kDa in the crude mitochondrial fraction that disappeared in the presence of E-64 (Figure 4d, arrow). Moreover, the E-64-sensitive band was undetectable when the sample was pre-heated.

**Figure 5.**
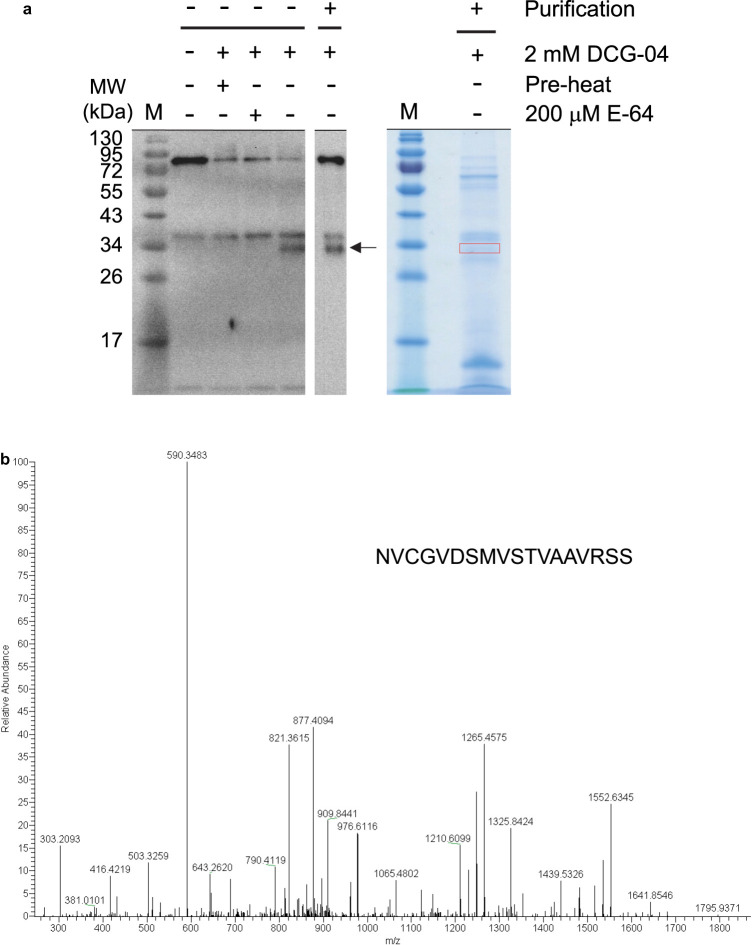
Identification of DCG-04-targeted protease in mitochondria from *A. maculatum*. (**a**) Purification of DCG-04-reactive proteins. Mitochondrial proteins were labelled with and without DCG-04 either in the presence or absence of E-64 as a competitor. A pre-heated sample (100°C, 5 min) was also labelled with DCG-04 (pre-heat). After labelling, the samples were purified using streptavidin beads. Samples were then separated on SDS–PAGE and analyzed by florescence scanning (left) and Coomassie Brilliant Blue staining (right). The DCG-04-reactive specific band is denoted by an arrow, and a band corresponding to the position of the DCG-04-reactive protein (indicated by a red box) was excised and analyzed by nano-LC–MS/MS. (**b**) MS/MS spectrum of the DCG-04-targeted protease peptide fragment (NVCGVDSMVSTVAAVRSS) derived by tryptic digestion of the protein sample.

The DCG-04-labelled proteins were next affinity purified, and the activity profiling of the mitochondrial proteins revealed the existence of a heat-labile, E-64-sensitive band at ∼30 kDa on the gel (Figure 5a). A band that corresponded to the specific DCG-labelled protein was excised and subjected to nano-LC–MS/MS analysis (Figure 5b). By MS/MS scanning and a MASCOT database search, a partial peptide (NVCGVDSMVSTVAAVRSS) that has been deposited as a cysteine protease 1-like protein from *Phoenix dactylifera* (NCBI Reference Sequence: XP_008775532.1) was found to be present in the mitochondria from *A. maculatum* (Figure 5b; Supplementary Figure S5; Supplementary Table S1).

## Discussion

Mitochondria harbour a proteolytic system that can specifically recognize and degrade proteins in this organelle. In general, peptidases in this system are highly conserved and can be classed into three groups: processing peptidases, ATP-dependent proteases and oligopeptidases [57]. The processing peptidases mediate limited proteolysis, whereas the ATP-dependent proteases and oligopeptidases, working sequentially, degrade proteins to single amino acids. Additionally, peptides generated by processing enzymes are degraded to amino acids by oligopeptidases [58]. Three families of ATP-dependent proteases have been identified in plant mitochondria: Lon, Clp and FtsH [57]. Lon and Clp are serine proteases, whereas the FtsH family are zinc-dependent metalloproteases. Although Lon proteases function in the control of protein turnover and/or protein complex assembly in plant organelles, there was no evidence previously that the expression patterns of nuclear genes encoding respiratory proteins, i.e. the cytochrome *c* 1, *cytc-2* genes and the *AOX1a* gene, were altered in a *lon1* mutant in comparison with wild type plants [59].

In our present study, we found that AOX proteins are degraded by an E-64-sensitive cysteine protease in the mitochondria of *A. maculatum*. Notably, this detected E-64-sensitive cysteine protease exerts its function at a higher temperature (30°C). The maximum temperature of the appendix reaches 32°C at the δ-stage during floral development in *A. maculatum* [15], and it was clear that such δ-stage-specific endogenous thermogenesis could greatly stimulate the protease activity that degrades the AmAOX proteins in mitochondria. Interestingly, the mitochondrial outer-membrane protein, VDAC, and mitochondrial matrix protein, Hsp60, were both almost intact under conditions where mitochondrial AmAOX proteins were degraded by the E-64-sensitive protease. Remarkably, the activities of the E-64-sensitive cysteine protease could be recovered from mitochondrial membrane fractions together with AmAOX proteins, and partial purification of this protease revealed a protein with an apparent molecular mass of 30 kDa that contains a peptide fragment that is identical with the C-terminal sequence of cysteine proteinase 1-like protein from *P. dactylifera* (Figure 5 and Supplementary Figure S5). Accordingly, our results strongly suggest that AmAOX proteins are the target of an as yet unidentified E-64-sensitive mitochondrial cysteine protease in thermogenic *A. maculatum*.

We conducted comparative expression analyses of *AmAOX* transcripts and their translation products using the same tissue samples collected at different developmental stages and thermogenic states (Figure 1a–c). Intriguingly, although the expression of *AmAOX* transcripts are greatly stimulated in thermogenic (hot) appendices, their protein translation levels remain unchanged across different developmental stages (pre-hot, non-hot, and hot appendices). These data are consistent with our previous paper which demonstrated that AmAOX proteins constitutively express at all stages during floral development in *A. maculatum* [13]. It should also be noted here that although mitochondria from non-hot and hot appendices both showed almost the same temperature-dependent protease activities for AOX proteins (Figure 2), the *AmAOX* transcript levels were significantly higher in the hot appendices (Figure 1). An important question arising from these findings is why there is a low correlation between the AOX mRNA and protein levels in the appendices. Presumably, the *AmAOX* transcript levels in pre-hot and non-hot appendices were sufficient to maintain the required amounts of AmAOX proteins in these tissues. In the hot appendices however, a much higher amounts of *AmAOX* transcripts is needed to maintain the translation products in the mitochondria at a level that is comparable to those in pre-hot and non-hot appendices. One of the possible mechanisms could be a temperature-dependent retrograde control pathway that links the increased degradation of AmAOX proteins in mitochondria and the stimulation of *AmAOX* transcription in the nuclei.

Organelle-to-nucleus signalling, or retrograde control, co-ordinates the expression of nuclear genes [60]. Mitochondrial retrograde regulation was first reported in *Saccharomyces cerevisiae* in which it was shown that perturbed respiratory function activates a retrograde pathway that regulates gene expression and thereby alters cellular metabolism [61]. The *AOX* genes represent the pre-eminent example of this system in plants [29,36,37,41]. In *A. thaliana,* the transcription of *AtAOX1a* has been shown to be strongly negatively regulated by ABI4 [38], but to be inducible by latent transcriptional factors including NAC17 [62,63]. *AtAOX1a* has also been shown to be under both negative and positive regulation by the WRKY transcription factors that bind to the *AtAOX1a* promoter [34,64,65]. Moreover, *AtAOX1a* transcription can be controlled by a number of regulators such as KIN10 that do not directly bind to its promoter [66]. Among these factors that directly or indirectly regulate *AtAOX1a* transcription, NAC17 is a tail-anchored protein in the endoplasmic reticulum that is released by rhomboid protease-mediated proteolytic cleavage to activate the expression of *AtAOX1a* and other downstream regulators of *AtAOX1a* [62]. Although the genomic sequencing of *A. maculatum* has not yet been completed and there is no information as to the promoter sequences of *AmAOXs,* we speculate that retrograde signalling pathways that control the transcription of *AtAOX1a* may also be involved in regulating the temperature-dependent gene expression of *AmAOXs* in *A. maculatum*. It is also possible that the temperature-dependent degradation of AmAOX proteins by the E-64-sensitive cysteine protease produces smaller peptide fragments that signal the activation of transcription (Supplementary Figure S6), as shown in *Caenorhabditis elegans* [67]. In our model, E-64-sensitivecysteine protease(s) are activated by an elevated appendix temperature caused by endogenous thermogenesis, which leads to an increased degradation of the AOX protein. Moreover, subsequent induction of *AOX* gene expression via unidentified signalling pathway to the nucleus could be activated (Supplementary Figure S6).

Ubiquinol is a common substrate for both AOX- and COX-mediated respiration pathways, and previous study showed that the ubiquinone pool in *A. maculatum* appendices was ∼90% reduced during thermogenesis [68]. Because a higher reduced ubiquinone pool provides maximal activity of the AOX-mediated respiratory pathway [13], it is clear that AmAOX proteins that are constitutively expressed in pre- and non-hot appendices show less enzymatic activities in *A. maculatum*. In the present study, we also confirmed our previous findings that mitochondria from pre-hot appendices express the ENV type of pyruvate-sensitive AOXs (AmAOX1a-d and f), whereas the QDT type of pyruvate-insensitive AOX (AmAOX1e) is principally expressed in mitochondria from non-hot and hot appendices [15]. It is tempting to speculate that developmental switch of the *AmAOXs* gene expression from pre-hot to hot appendices is a ‘switching out’ of the pyruvate-sensitive AOX form for a pyruvate-insensitive AOX form. In this case, activities of pyruvate insensitive AmAOX1e might then no longer be dependent upon the threshold level of pyruvate concentration within the matrix of mitochondria in thermogenic appendices of *A. maculatum*.

The significance of increased AmAOX protein turnover in *A. maculatum* is a fundamental question to arise from our current data. AOX proteins in plant mitochondria generally exist as either a non-covalently associated reduced dimer (active form) or an oxidized dimer (inactive form) through the formation of a disulfide bridge between the conserved Cys I residue [22,69]. The ratio of reduced and oxidized AOX proteins at various equilibrium conditions could be estimated by computer simulation modelling and it is apparent that an elevated turnover rate of the AOX proteins reduces the oxidized state level (Supplementary Figure S7). Although the oxidative damage of mitochondrial proteins, which is induced under higher respiration conditions, leads to structural alterations and further degradation by specific proteases [55,70], an increased turnover of AOX proteins, which is stimulated by endogenous thermogenesis, could also have a role to preserve AmAOX proteins in a reduced (active) form in thermogenic appendices of *A. maculatum*.

In conclusion, AOX proteins are a potential substrate of an E-64-sensitive cysteine protease of the thermogenic appendices of *A. maculatum*. The temperature-inducible degradation of AOX proteins and the subsequent stimulation of their gene transcription may contribute to the endogenous heat-production in this plant.

## Abbreviations

AEBSF: 4-(2-Aminoethyl) benzenesulfonyl fluoride hydrochloride
AOX: alternative oxidase
BCA: bicinchoninic acid
CBB: Coomassie Brilliant Blue
COX: cytochrome *c* oxidase
DNPH: 2, 4-dinitrophenylhydrazine
PVDF: polyvinylidene difluoride
qRT-PCR: quantitative real time-PCR
ROS: reactive oxygen species
SDS–PAGE: sodium dodecyl sulfate-polyacrylamide gel electrophoresis
VDAC: voltage-dependent anion channel
