# Supplementary material for: Degradation of mitochondrial alternative oxidase in the appendices of Arum maculatum
Source: Biochem J. 2020 Sep 17;477(17):3417–31. doi: 10.1042/BCJ20200515 (PMC7505559; doi:10.1042/BCJ20200515)
Supplement: Supplementary Figures S1-S7 and Table S1 [file BCJ-477-3417-s1.pdf]

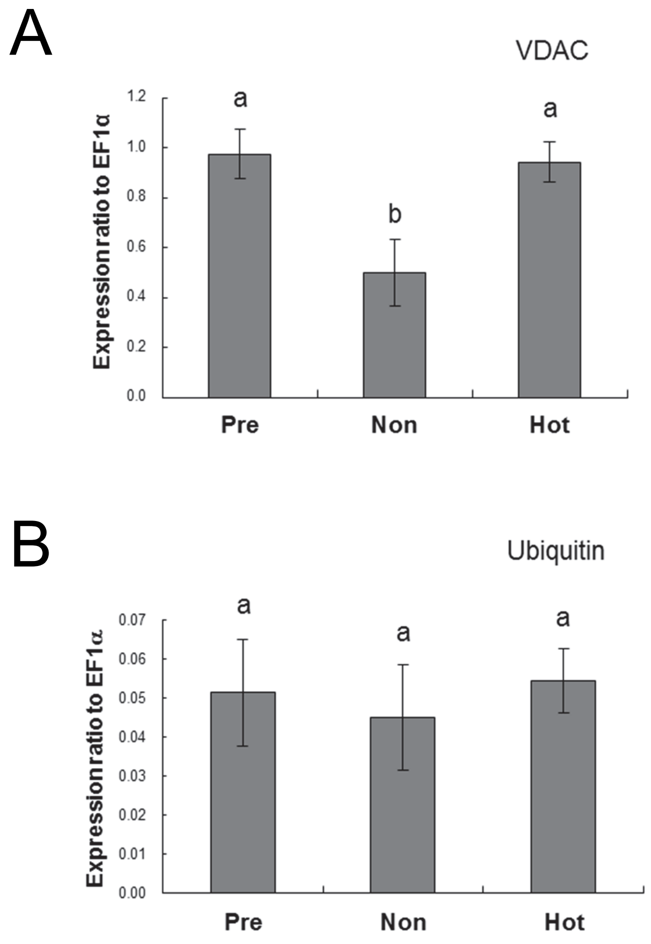

Supplemental Figure S1.

Expression of *VDAC* and *ubiquitin* transcripts analysed by qRT-PCR in various thermogenic stage appendices of *A. maculatum*. A, Expression levels of *VDAC* transcripts shown as a ratio relative to *EF1a*. B, Expression levels of *ubiquitin* transcripts analysed by qRT-PCR depicted as an expression ratio relative to *EF1a*. Different alphabetical letters in the graph indicates significantly different values (n = 3, P < 0.05).

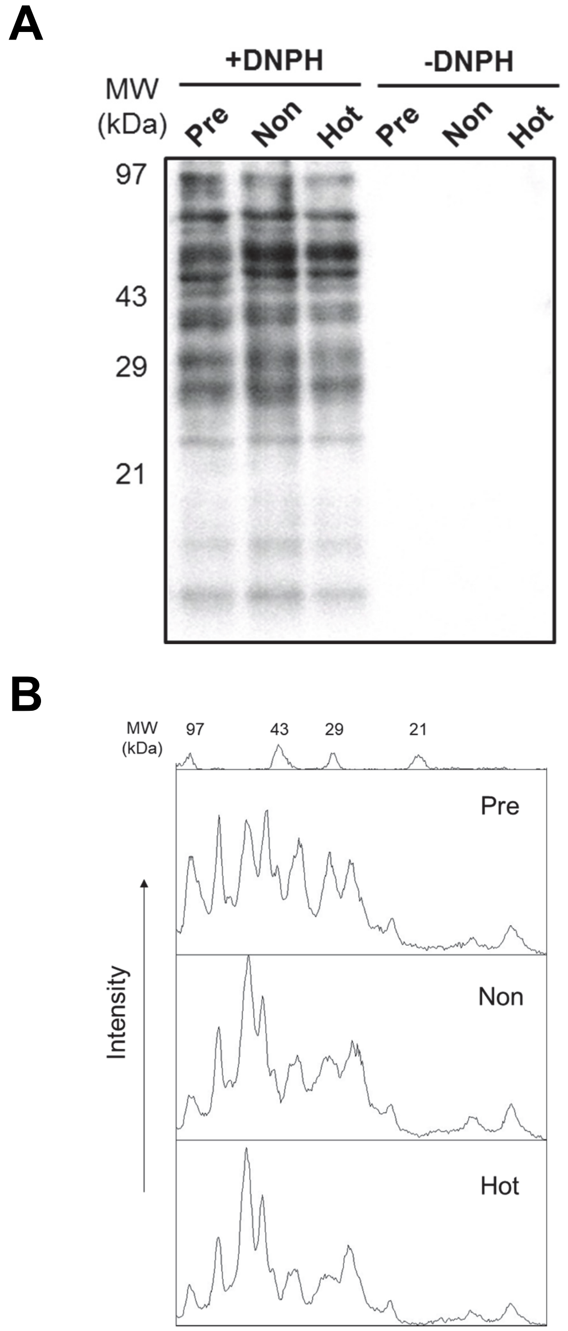

Supplemental Figure S2.
Detection of carbonylated proteins in mitochondria purified from appendices at various developmental stages in *A. maculatum*. A, Four micrograms of DNPH-treated (+DNPH) or untreated (−DNPH) mitochondrial proteins were resolved by SDS-PAGE and blotted onto a polyvinylidene difluoride membrane. DNP-modified proteins were detected immunochemically as described in the Materials and Methods. The molecular mass standards are indicated on the left. B, Densitometric analysis of DNPH-positive (+DNPH) signals shown in panel A.

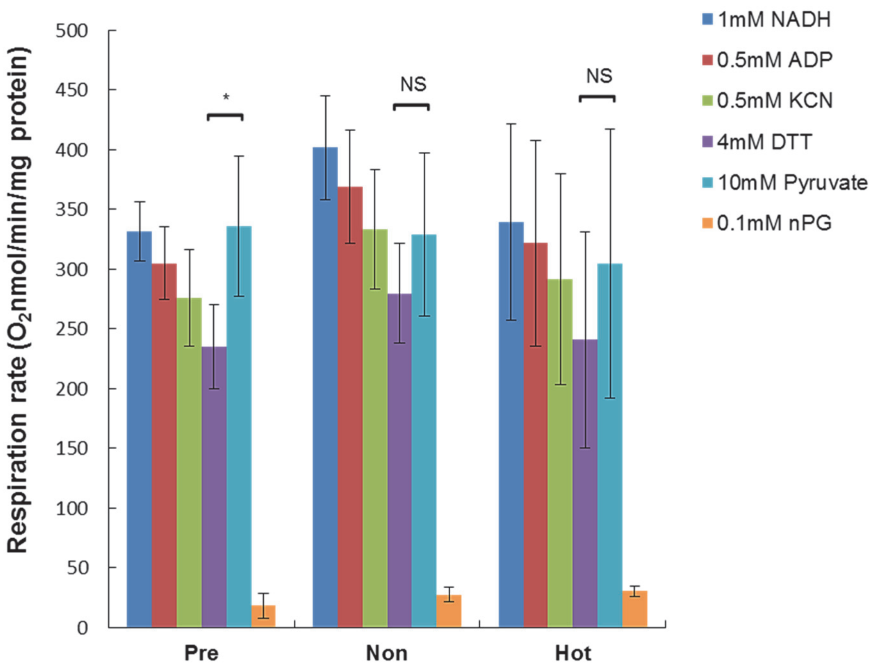

Supplemental Figure S3.

Respiration rates in mitochondria purified from appendices at various developmental stages in *A. maculatum*. Rates of oxygen uptake were determined using an oxygen electrode after sequential addition of the compounds at the final concentrations shown in the graph. NS: not significant. Data are the means ± SD (n = 3).

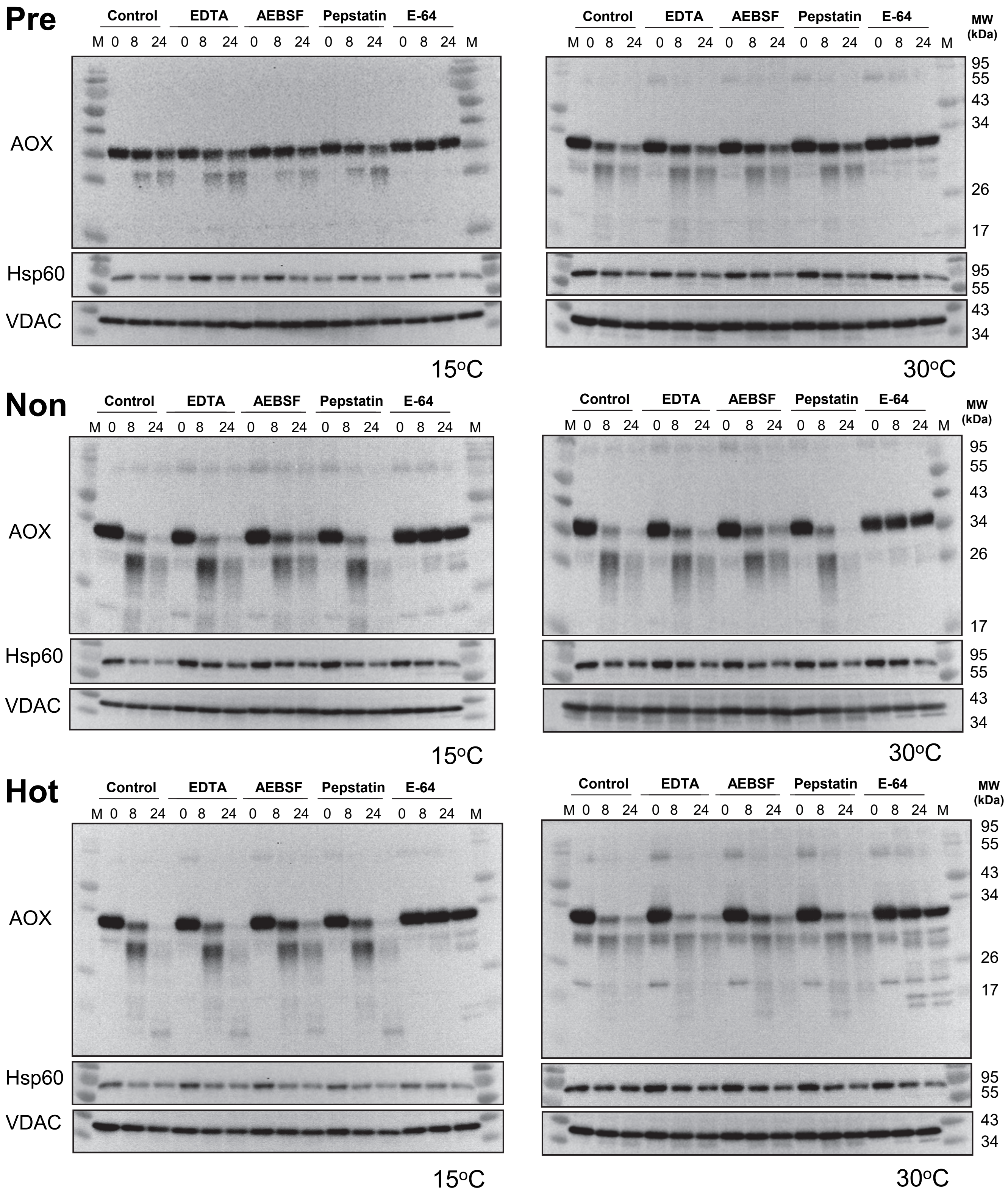

Supplemental Figure S4.

Effects of proteinase inhibitors on the degradation of AmAOX, Hsp60 and VDAC proteins in mitochondria purified from appendices at various developmental stages in *A. maculatum*.

Mitochondrial proteins (40 μg) were incubated at either 15°C or 30°C in a buffer containing 200 mM Tris-HCl, pH 7.5, 10 mM $MgCl_2$, 10 mM $CaCl_2$, 50 mM ATP and 0.1% Triton-X100 for the indicated time periods (0, 8 and 24 h). EDTA, AEBSF, pepstatin and E-64 were added at final concentrations of 5 mM, 1 mM, 1 μM and 28 μM, respectively. Samples were separated by SDS-PAGE, transferred to a polyvinylidene difluoride membrane and incubated with antibodies against AOX, Hsp60 and VDAC. Molecular mass standards are indicated.

```
  1 MDRRFVLLLL  LVGVALPASV  ASSSPSDADD  DPLIVQVVSE  ADEDELMLNA
 51 EAHFSSFLRR  FGKSYADEKE  HAYRFSVFKA  NLRRARRHQK  MDPTAVHGIT
101 KYSDLTPAEF  RRTFLGLRGG  RRLRRALASS  HEAPILPTND  LPTDFDWRDH
151 GAVTGVKDQG  SCGSCWSFSA  AGSLEGANFL  ATGKLESLSE  QQLVDCDHEC
201 DSSEPDSCDS  GCNGGLMTTA  FEYLLKSGGL  EREEDYPYTG  TDRGRCKFDK
251 SKIAASVKNF  SVVSMDEDQI  AANLVKHGPL  AVGINAVFMQ  TYIGGVSCPY
301 ICGRHLDHGV  LLVGYGSAAY  APIRFKEKPY  WIIKNSWGEK  WGENGYYKIC
351 RGRNVCGVDS  MVSTVAAVRS  S
```

Supplemental Figure S5.
Amino acid sequence of the predicted cysteine proteinase 1-like protein from *Phoenix dactylifera* (NCBI Reference Sequence: XP_008775532.1). An identical amino acid sequence obtained from a MASCOT database search is indicated in red lettering.

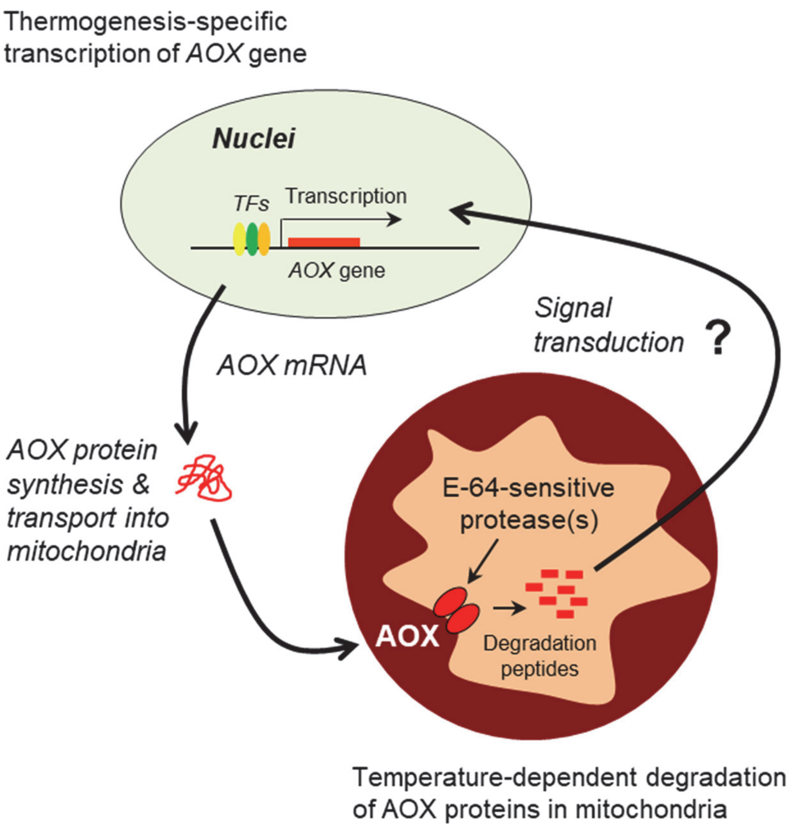

Supplemental Figure S6.

A model for AOX turnover and possible retrograde signalling in the thermogenic appendices of *A. maculatum.* Degradation of the AOX protein is induced by E-64-sensitive cysteine protease(s) whose activity is stimulated by an elevated appendix temperature caused by endogenous thermogenesis. A hypothetical signalling pathway to the nucleus and the subsequent induction of *AOX* gene expression are shown. TFs, transcription factors.

A

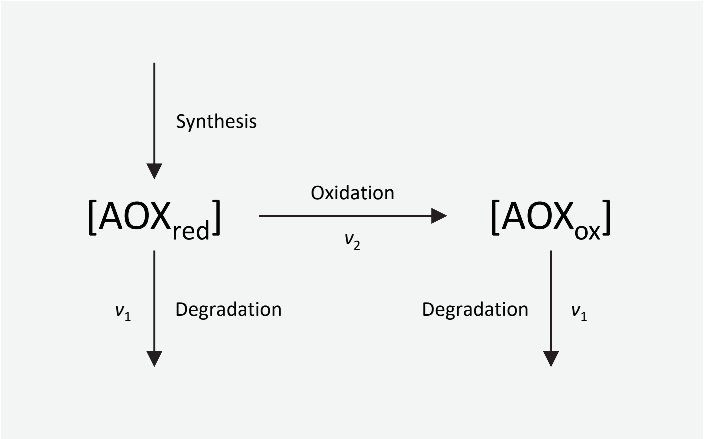

B

$$\begin{cases} \dfrac{d[AOX_{red}]}{dt} = v_1[AOX_{ox}] - v_2[AOX_{red}] = 0 \\ \dfrac{d[AOX_{ox}]}{dt} = v_2[AOX_{red}] - v_1[AOX_{ox}] = 0 \end{cases}$$

$$[AOX_{red}] = \frac{v_1}{v_2}[AOX_{ox}]$$

$$\frac{[AOX_{ox}]}{[AOX_{red}] + [AOX_{ox}]} = \frac{[AOX_{ox}]}{\frac{v_1}{v_2}[AOX_{ox}] + [AOX_{ox}]} = \frac{1}{\frac{v_1}{v_2} + 1}$$

C

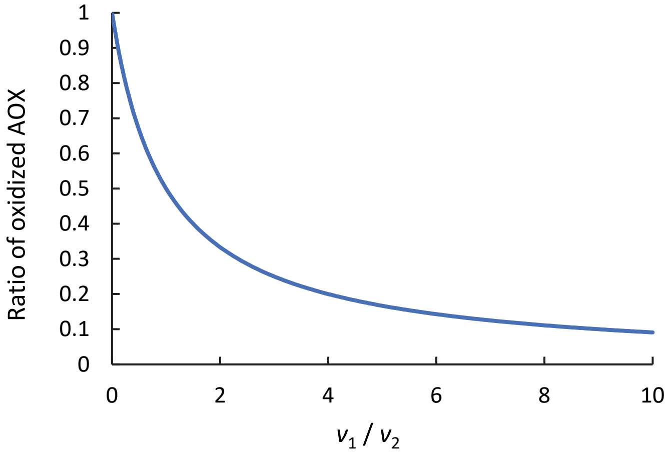

Supplemental Figure S7.

Modelling and computer simulation of AOX turnover in the appendices of *A. maculatum*. A. Turnover of AOX proteins. Reduced AOX proteins ($[AOX_{red}]$) and oxidized AOX proteins ($[AOX_{ox}]$) are degraded at a rate of v_1. $[AOX_{red}]$ are oxidized at a rate of v_2. B. Ratio of $[AOX_{red}]$ and $[AOX_{ox}]$ at equilibrium shown in panel A. C. Relationship between the ratio of oxidized AOX proteins and turnover rates represented by v_1 / v_2.

Supplemental Table S1.
Mass of the amino acid fragment ions from the tryptic peptide NVCGVDSMVSTVAAVRSS.

No.	b	b^{++}	Amino Acid	y	y^{++}	No.
1	115.0502	58.0287	N			18
2	214.1186	107.5629	V	1740.8255	870.9164	17
3	374.1493	187.5783	C	***1641.7571***	**821.3822**	16
4	431.1707	216.0890	G	1481.7264	741.3669	15
5	530.2391	265.6232	V	***1424.7050***	712.8561	14
6	645.2661	323.1367	D	***1325.6366***	663.3219	13
7	***732.2981***	366.6527	S	***1210.6096***	605.8084	12
8	879.3335	440.1704	M	***1123.5776***	562.2924	11
9	***978.4019***	489.7046	V	***976.5422***	488.7747	10
10	***1065.4340***	533.2206	S	***877.4738***	439.2405	9
11	1166.4816	583.7445	T	***790.4417***	395.7245	8
12	***1265.5501***	633.2787	V	***689.3941***	345.2007	7
13	1336.5872	668.7972	A	***590.3257***	295.6665	6
14	1407.6243	704.3158	A	519.2885	260.1479	5
15	1506.6927	753.8500	V	448.2514	224.6293	4
16	1662.7938	831.9005	R	349.1830	175.0951	3
17	1749.8258	875.4166	S	193.0819	97.0446	2
18			S	106.0499	53.5286	1

Bold italic red means the series contributed to the score. Bold red means that the number of matches in the ion series is greater than would be expected by chance.
